# ASSOCIATIONS BETWEEN PERSONALITY TRAITS AND COGNITIVE RESILIENCE IN OLDER ADULTS

**DOI:** 10.1093/geroni/igz038.2861

**Published:** 2019-11-08

**Authors:** Bryan D James, Daniel K Mroczek, Eileen K Graham

**Affiliations:** 1 Rush Alzheimer’s Disease Center, Chicago, Illinois, United States; 2 Northwestern University, Chicago, Illinois, United States

## Abstract

There is often discordance between brain pathology and dementia diagnosis. Some individuals maintain cognitive function throughout their lives but show high burden of neuropathology after death (e.g. amyloid plagues, neurofibrillary tangles, vascular disease, Lewy bodies, and/or TDP-43). Conversely, some demonstrate significant decline and receive a dementia diagnosis, while showing minimal neuropathology at autopsy. The current study seeks to understand these resilience/vulnerability profiles, with a focus on individual differences. That is, are individuals with certain personality characteristics (e.g. high openness, low neuroticism) more/less likely to have cognitive resilience or vulnerability? Using psychosocial and autopsy data from the Rush Memory and Aging Project and the Religious Orders Study, this study uses a resilience index based on residuals derived from regressing global cognition on global pathology, then entering personality traits as predictors of cognitive resilience. The analysis plan will be submitted to the Journal of Gerontology’s special issue on pre-registration of existing data.

